# Review and New Perspectives on Non-Layered Manganese Compounds as Electrode Material for Sodium-Ion Batteries

**DOI:** 10.3390/ma16216970

**Published:** 2023-10-30

**Authors:** Ricardo Alcántara, Carlos Pérez-Vicente, Pedro Lavela, José L. Tirado, Alejandro Medina, Radostina Stoyanova

**Affiliations:** 1 Department of Inorganic Chemistry, Institute of Chemistry for Energy and Environment (IQUEMA), Faculty of Sciences, Campus of Rabanales, University of Cordoba, Building Marie Curie, 14071 Córdoba, Spain; iq3pevic@uco.es (C.P.-V.); iq1lacap@uco.es (P.L.); iq1ticoj@uco.es (J.L.T.); q42mejaa@uco.es (A.M.); 2 Institute of General and Inorganic Chemistry, Bulgarian Academy of Sciences, 1113 Sofia, Bulgaria; radstoy@svr.igic.bas.bg

**Keywords:** post-lithium batteries, sodium-ion batteries, spinel, phosphate, multianion, manganese compounds

## Abstract

After more than 30 years of delay compared to lithium-ion batteries, sodium analogs are now emerging in the market. This is a result of the concerns regarding sustainability and production costs of the former, as well as issues related to safety and toxicity. Electrode materials for the new sodium-ion batteries may contain available and sustainable elements such as sodium itself, as well as iron or manganese, while eliminating the common cobalt cathode compounds and copper anode current collectors for lithium-ion batteries. The multiple oxidation states, abundance, and availability of manganese favor its use, as it was shown early on for primary batteries. Regarding structural considerations, an extraordinarily successful group of cathode materials are layered oxides of sodium, and transition metals, with manganese being the major component. However, other technologies point towards Prussian blue analogs, NASICON-related phosphates, and fluorophosphates. The role of manganese in these structural families and other oxide or halide compounds has until now not been fully explored. In this direction, the present review paper deals with the different Mn-containing solids with a non-layered structure already evaluated. The study aims to systematize the current knowledge on this topic and highlight new possibilities for further study, such as the concept of entatic state applied to electrodes.

## 1. Introduction

**Figure 1:**
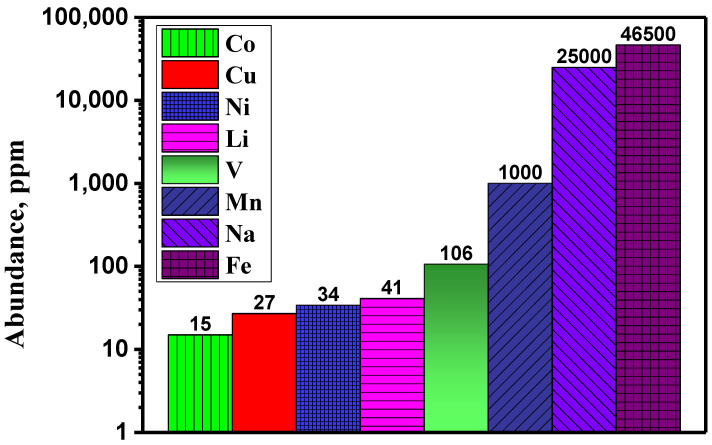
Element abundances of the upper continental crust [1,2].

Rechargeable batteries for renewable energy storage should be made from abundant, inexpensive, and low-toxicity elements. The production of lithium-ion batteries could be limited mainly due to the scarcity of mineral reserves and the high cost of lithium and other elements such as cobalt, nickel, and copper (Figure 1) [1,2]. Therefore, abundant, and cheap materials for sustainable batteries are being intensively investigated. SIB could be competitive against LIB, particularly in terms of economic cost and abundance of mineral resources. Another advantage is that aluminum could be used as a current collector for both the positive and the negative electrode because Na does not alloy with Al, avoiding Cu. In addition, SIB can be particularly useful for large-scale energy storage.

Unfortunately, the larger size of Na^+^ compared to Li^+^ (1.02 vs. 0.69 Å) could be a disadvantage, and the accommodation and mobility of sodium into the host material can be difficult. The structural changes induced by the intercalation and deintercalation of sodium can deteriorate the crystal structure and lead to battery failure. Thus, finding the most suitable materials for SIB is a great challenge.

Manganese is particularly interesting as the main component of the electrode active material, because of its low cost, natural abundance (Figure 1), and low toxicity, compared to other elements such as nickel and cobalt. Early investigations unveiled the existence of several phases in the Na_x_MnO_2_ system (0 < x < 1) in which sodium ions can be (de)intercalated [3,4]. Thus, several manganese oxides are promising as electrode-active materials for SIB, including 3D and 2D structures. Another advantage could be the diversity of oxidation states of Mn, which could help deliver high capacity. The study and selection of the most adequate structures are key for developing high-performance electrodes for non-aqueous sodium-ion batteries. A main challenge for using some manganese oxides, such as the spinel-type compound AMn_2_O_4_, is the instability of the crystal structure because the structural change can drive battery failure. Many layered oxides (2D structures) are often incapable of accommodating the structural change and the strains due to the Jahn–Teller effect of Mn(III) ion during the charge/discharge of the battery.

**Figure 2:**
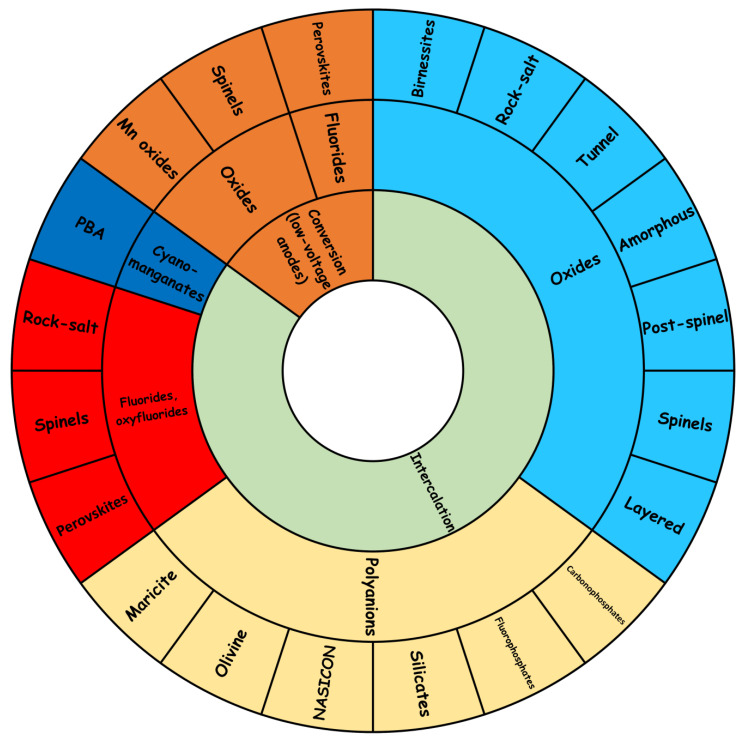
Schematic overview of the main types of Mn-based materials for SIB.

The low cost, natural abundance, and sustainability of sodium and manganese elements are the main justifications for the interest in these materials. Although we have learnt a lot from LIBs, SIBs are still more challenging, and great efforts are still needed. The layered-type oxides are not included in this review, except for the heterostructured materials, because the layered-type materials have been extensively investigated and the great number of papers published on this subject would deserve another review paper. A main disadvantage of the layered-type materials is that often they suffer structure transformation and poor cycling stability. Some non-layered materials could be competitive in terms of cycling stability. The main goal of this article is to review the main properties of these materials, their advantages and disadvantages, and the challenges and possible ways to advance in this field. This review first focuses on Mn-based oxides with spinel-type structures and other structures related to that such as tunneled-type, and post-spinel, which are employed as intercalation electrode material in non-aqueous sodium-ion batteries. Secondly, manganese fluorides and oxyfluorides are reviewed. Thirdly, the most relevant manganese-based multianion compounds, such as phosphates, carbonates, and silicates, are also included. Finally, the conversion electrode materials are reviewed. The classification of these types of materials is schematized in Figure 2, and the main properties of the materials are summarized in Table 1.

## 2. Manganese Oxides for Sodium Intercalation

For the intercalation of sodium into manganese oxides at voltages over ca. 2 V vs. Na^+^/Na, compounds with a variety of structures have been studied, such as spinel, post-spinel, tunnel, and rock salt, and these compounds are reviewed below.

### 2.1. Spinel-Type NaMn_2_O_4_

**Figure 3:**
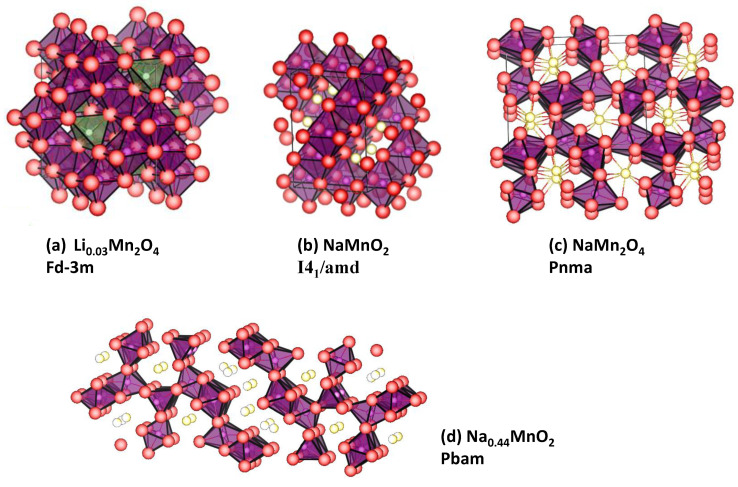
Structures of several Mn-containing electrode materials. (**a**) Cubic spinel Li_0.03_Mn_2_O_4_ [16]. (**b**) Tetragonal spinel NaMnO_2_ [13]. (**c**) NaMn_2_O_4_ [17]. (**d**) Tunnel-type, Na_0.44_MnO_2_ [18].

The synthesis of spinel-type NaMn_2_O_4_ using the conventional solid-state method is impossible. Tarascon et al. obtained cubic λ-MnO_2_ (Figure 3) by following the procedure reported by Hunter, which is based on the delithiation of the spinel-type LiMn_2_O_4_ by acid-leaching or by chemical oxidation, and then they found that the electrochemical insertion of sodium into λ-MnO_2_ drives the migration of manganese atoms in the oxide framework and structure transformation from spinel-type to layered-type Na_x_MnO_2_ (with Δx = 0.6) [5,6].

As an alternative to cubic λ-MnO_2_, Bach et al. employed the tetragonal spinel Mn_2.2_Co_0.27_O_4_ [7]. Up to one Na per formula unit (NaMn_2.2_Co_0.27_O_4_) can reversibly intercalate in the 3D structure. However, the capacity retention was not particularly good, and around 0.67–0.5 Na intercalated after 10 cycles.

It is generally accepted that the spinel-type NaMn_2_O_4_, with 3D channels for Na-diffusion, is not thermodynamically stable [8,9]. Thus, the spinel transforms into the layered form of NaMn_2_O_4_ after a few cycles in a sodium battery. Inversely, the spinel LiMn_2_O_4_ is more stable than the layered form of Li_0.5_MnO_2_, and the spontaneous transformation of the layered structure into a spinel structure was observed during cycling in lithium batteries [10]. This is related to the higher energy barrier for manganese migration and cation mixing in the oxide packing of layered O3 Na_0.5_MnO_2_ compared to Li_0.5_MnO_2_, because manganese migration involves a difficult displacement of sodium to the tetrahedral site.

Yabuuchi et al. confirmed that the electrochemical delithiation and then sodium insertion into stoichiometric LiMn_2_O_4_ (s.g. Fd-3m) induce a phase transition to layered NaMnO_2_. This layered NaMnO_2_ exhibits relatively good capacity retention and ca. 130 mAh g^−1^ of capacity for a voltage range between 3.0 and 4.3 V, and the capacity is higher (190 mAh g^−1^), but the retention is poorer for the 2.3–4.3 V voltage range. They also reported that the intercalation of sodium into non-stoichiometric spinel-type Li[Li_0.2_Mn_1.8_]O_4_ induces strain in the crystal lattice and the phase transition into the layered phase was not observed [11].

Tang et al. delithiated the spinel-type LiMn_2_O_4_ by charging up to 4.3 vs. Li^+^/Li, and then sodiated the resulting manganese oxide by discharging down to 2.0 V vs. Na^+^/Na [12]. The resulting sample is a mixture of spinel-type NaMn_2_O_4_ and layered Na_x_Mn_2_O_4_ with a small amount of residual lithium. Interestingly, the in situ formed layered structure appears as a shell surrounding the core spinel structure in a single particle forming and intergrowth structure. In this spinel-layered intergrowth structure, the layered phase would be the main phase for the reversible intercalation of sodium, while the spinel phase would stabilize the electrode material during the charge/discharge process.

Instead of using LiMn_2_O_4_, Kataoka et al. first prepared monoclinic layered Li_2_Mn_2_O_3_ (s.g. C2/m) as a precursor [13]. Secondly, they obtained Li_2−x_MnO_3_ with cubic spinel structure (s.g. Fd-3m) by electrochemical delithiation of Li_2_Mn_2_O_3_ and rinsing several times with dimethyl carbonate solvent. Finally, they used the resulting delithiated manganese oxide with x = 1.6–1.8 in a sodium cell, with an experimental capacity of around 160–200 mAh g^−1^. The resulting electrode material retains the spinel structure during sodiation/desodiation cycles, and they suggested that the migration of Mn to the tetrahedral sites is more energetically unfavorable than for the lithiated compound, due to the difference in ionic radii, and the cycle stability can be better in sodium than lithium cell. In addition, they pointed out that the structure of this spinel NaMnO_2_ is tetragonally distorted (Figure 3).

For the AMn_2_O_4_ spinel (A = Li, Na or Mg), Kolli and Van der Ven assumed that small cations (Li^+^ and Mg^2+^) prefer to occupy the tetrahedral site, while larger Na^+^ prefers to occupy the octahedral site (16c) [14]. Recent theoretical calculations on the intercalation of sodium into λ-MnO_2_ unveiled that initially the occupation of the tetrahedral site by sodium is energetically more favorable than the octahedral site for the composition Na_0.125_Mn_2_O_4_ [15]. The calculated insertion voltage is 2.85 V, and the lattice cell would expand upon sodium intercalation. On the other hand, this result does not involve that the sodiated spinel is thermodynamically stable, and in fact, it is known that more sodiation yields the layered structure. In fact, the structural transformation of the spinel can be detrimental to the electrochemical cycling.

### 2.2. Calcium Ferrite-Type NaMn_2_O_4_

In 2006, Yamamura et al. reported that the spinel-type structure of LiMn_2_O_4_ can be transformed into the CaFe_2_O_4_-type by heating under 6 GPa of pressure, and thus a new form of LiMn_2_O_4_ was obtained [19]. This calcium ferrite structure is about 6% denser than the spinel, all the Mn are in a 6-fold coordination by oxygen, and the MnO_6_ forms a double chain-type unit.

Analogously to LiMn_2_O_4_, the so-called “post-spinel” NaMn_2_O_4_, with CaFe_2_O_4_-type structure and s.g. Pnma was first obtained by the structural transformation of spinel NaMn_2_O_4_ under high pressure by Akimoto et al. [17] (Figure 3). The MnO_6_ octahedra share edges and form 1D tunnels for easy sodium diffusion. In contrast to the spinel-type NaMn_2_O_4_, the post-spinel NaMn_2_O_4_ is thermodynamically stable. The high energy barrier of the rearrangement of the MnO_6_ octahedron suppresses the Jahn–Teller distortion in the post-spinel NaMn_2_O_4_. Unfortunately, the reinsertion of sodium is very difficult, particularly for large particles [20].

The post-spinel type NaMnSnO_4_ (s.g. Pnma), in which tin atoms stabilize the structure, can be synthesized at ambient pressure [21]. The reversible capacity of this material in a sodium cell was only about 25–30 mAh g^−1^ in the range between 2.0 and 4.5 V, and the XRD patterns do not show significant changes during sodium (de)intercalation.

### 2.3. Tunnel-Type Na_0.44_MnO_2_

**Figure 4:**
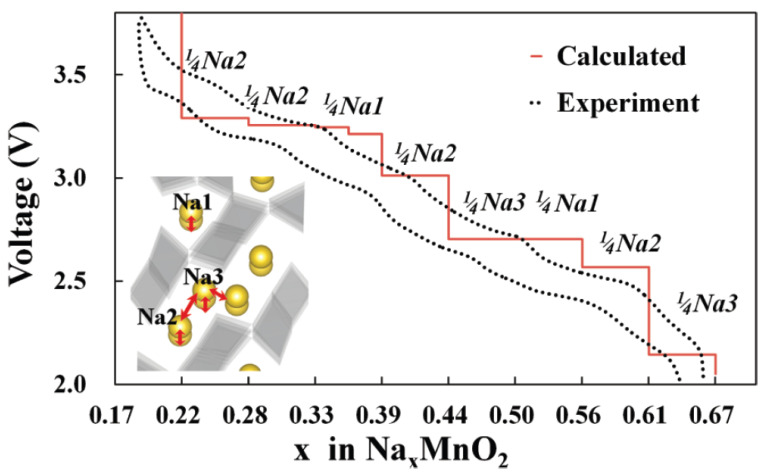
Calculated and experimental voltage profile of tunnel-type Na_x_MnO_2_ in sodium cell. Reprinted (adapted) with permission from Ref. [25]. Copyright (2012) ACS.

Doeff et al. first reported the use of orthorhombic Na_x_MnO_2_ with x = 0.44 and 0.2 as the cathode of a sodium battery at 85 °C and they employed poly(ethylene oxide) and NaCF_3_SO_3_ in the polymer electrolyte [22]. They treated the compound Na_0.44_MnO_2_ with aqueous hydrochloric acid for its partial desodiation from x = 0.44 to 0.2. Later, Sauvage et al. prepared single-phase Na_4_Mn_9_O_18_ (or Na_0.44_MnO_2_) and provided a detailed structure characterization [23]. This oxide has an orthorhombic tunnel-type structure (s.g. Pbam) with two types of tunnels: a small tunnel and an S-shaped large tunnel. MnO_6_ and MnO_5_ polyhedrons form S-shaped tunnels, which are very suitable for sodium diffusion [23,24] (Figure 2). According to theoretical calculations, there are three sites for sodium (Na1, Na2, and Na3) [25]. The small tunnel is almost filled by Na1. A large S-shaped tunnel is half-filled by Na2 (trigonal prismatic coordination) and Na3. Due to the Jahn–Teller distortion of Mn^3+^, the change of the lattice cell parameters in crystallographic directions of the b-axis and c-axis is anisotropic. Interestingly, the transformation to the spinel-type structure does not occur due to a size mismatch between Na and Mn and different oxygen frameworks. The maximum capacity is 140 mAh g^−1^, and the system Na_x_MnO_2_ is fully reversible for 0.25 < x < 0.65 within the voltage range between 2.0 and 3.8 V (Figure 4) [23,25]. However, there is partial irreversibility for x < 0.25. The Jahn–Teller effect, the presence of several biphasic transitions, and the dissolution of Mn(II) ions into the electrolyte solution can be the main reasons for the poor capacity retention. If the amount of sodium is higher than x > 0.44, a layered structure may form.

A mixture between Mn(III) and Mn(IV) oxidation states is expected in Na_0.44_MnO_2_. Replacing the Jahn–Teller ion Mn(III) with a non-Jahn–Teller ion (Mn(IV)) can improve the cyclability [26,27]. Another strategy proposed by Liang et al. is Li-doping and the formation of a Na_0.44_MnO_2_/LiMn_2_O_4_ heterostructure [24]. The lithium ions in the heterostructure function as pillars to stabilize the structure.

Partial replacement of Mn by other transition metals is an explored strategy to enhance the electrochemical performance. Doping sodium manganate opens different opportunities. Ti-substituted Na_0.44_Mn_1−x_Ti_x_O_2_ also has a tunnel-type structure and has been a proposed electrode for both aqueous and non-aqueous sodium-ion batteries [28,29]. The incorporation of Ti results in an expansion of the unit cell volume. During the charge/discharge cycling, the composition changes between Na_0.22_Mn_1−x_Ti_x_O_2_ and Na_0.66_Mn_1−x_Ti_x_O_2_. Multiple plateaus appear in the voltage curve for Na_y_MnO_2_, while a sloping profile is observed for Ti-doped samples, which is typical of a solid solution. The disordered Mn/Ti arrangement could break the ordering in Na_0.66_Mn_1−x_Ti_x_O_2_ during the (de)intercalation of sodium. After the incorporation of Ti, the tunnel structure remains preserved even for higher Na-content, and the capacity retention is improved [30]. Similarly, according to Jia et al., Ti-doping particularly stabilizes the tunnel structure for Na_0.66_Mn_0.9_M_0.1_O_2+δ_ (M = dopant), the Ti-doped sample operates at higher voltage, and the observed hysteresis in the voltage curve minimizes [31].

Iron is another cheap and abundant element that could be employed in electrodes. The structure of Na_0.61_[Mn_0.61−x_Fe_x_Ti_0.39_]O_2_ is also tunnel-type. and these compounds are particularly air-stable [32]. According to XANES and ^57^Fe Mössbauer results, during the charge/discharge process, the oxidation state of manganese and iron (Fe^3+^/Fe^4+^) take part in the charge compensation, while the oxidation state of titanium remains unchanged. The participation of the iron redox couple provides a high average voltage (3.56 V).

For stabilization of the tunnel structure and preventing the transformation to a layer-type structure after cobalt-doping, it is necessary to keep the mole ratio Co/Mn under 0.01. For example, Zhong et al. studied Na_0.44_Mn_0.9925_Co_0.0075_O_2_ [33]. Since Co^3+^ is smaller than Mn^3+^, the MO_5_ and MO_6_ polyhedra contract, the S-shape tunnel is enlarged, and sodium mobility is easier. In addition, Co-doping improves the electronic conductivity and structural stability, and it could suppress the Mn dissolution. The incorporation of aluminum in Na_0.44_MnO_2_ drives a mixture between tunnel-type Na_0.44_MnO_2_ and orthorhombic layered-type NaAl_0.1_Mn_0.9_O_2_ [34]. The Al-O bonds in the surface of the particles contribute to improving the electrode stability.

**Figure 5:**
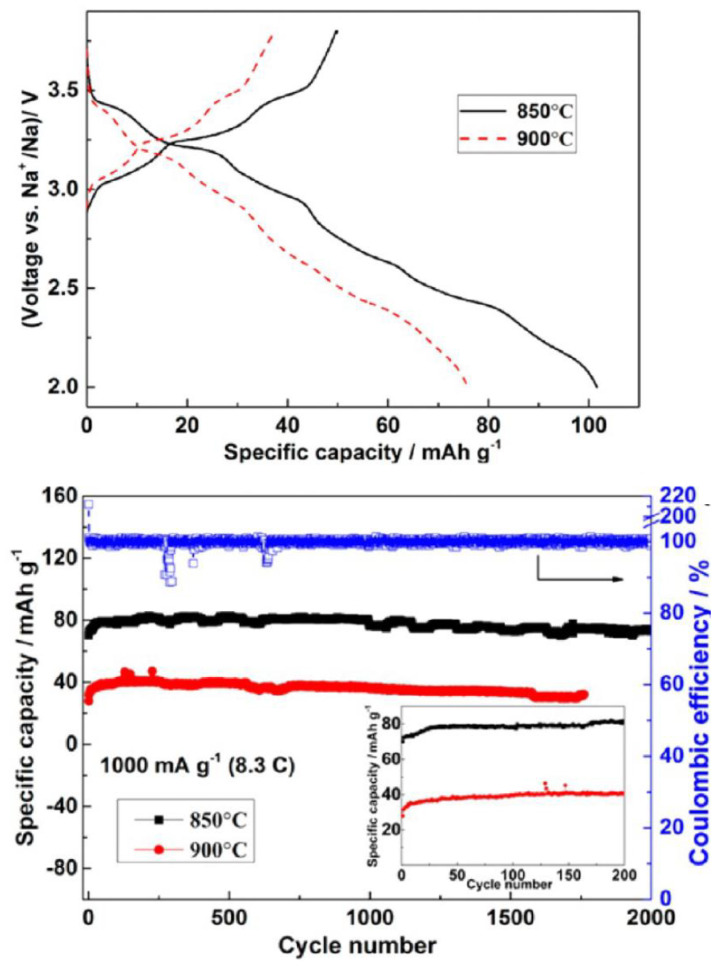
Electrochemical properties of multiangular rod-shaped Na_0.44_MnO_2_ in a sodium cell. Reprinted (adapted) with permission from Ref. [36]. Copyright (2017) ACS.

Control of the particle size and morphology can be another strategy to improve the charge/discharge rate and stability of Na_0.44_MnO_2_ [35,36,37]. For that purpose, Dai et al. employed a synthesis method based on the combustion of PVP and obtained rod-shaped particles [37]. The resulting diffusion coefficient of sodium was between 1.5 × 10^−^^12^ cm^2^ s^−1^ and 2.7 × 10^−^^10^ cm^2^ s^−1^, depending on the charge state, such as lithium in LiMn_2_O_4_. The maximum capacity value was 123 mAh g^−1^ at C/5. The direction [001] (parallel to the c-axis) is the favorite direction for the crystal growth in Na_0.44_MnO_2_ and then particles with nanorod morphology are easily prepared [38]. Nevertheless, the particles with nanoplate shape, which exhibit more particle surface perpendicular to the c-axis, reduce the distance of the more difficult pathway for sodium diffusion, and it can drive to a better rate capability. The reduction of the crystal growth in the [001] direction can provide outstanding high-rate capability (96 mAh g^−1^ at 10C) and remarkable cycling stability. Multiangular rod-shaped particles were prepared by using the reverse microemulsion method, and this material possesses very stable cycling performance (99.6% capacity retention after 2000 cycles), although the capacity is low (Figure 5) [36].

A green battery can be eco-friendly and developed with Na_0.44_MnO_2_ as a cathode, hard carbon as an anode, and CMC as a binder [39]. This CMC binder is low cost, not toxic, and it can be employed through aqueous processing, which improves sustainability and decreases the environmental impact of the battery technology, compared to processing with organic solvents. Interestingly, Na_0.44_MnO_2_ is sufficiently stable for aqueous processing. On the other hand, the hard carbon must be presodiated to compensate for its initial irreversible consumption of sodium, thus avoiding employing an excess of Na_0.44_MnO_2_. Another way to counteract the sodium deficiency of this cathode avoiding anode presodiation is by blending a sacrificing additive in the cathode, for example, pentasodium diethylenetriaminepentaacetate [40]. The urea-based solution combustion synthesis can be an eco-friendly route to obtain Na_0.44_MnO_2_ [41].

A multifunctional effect of Na_2_MoO_4_ is that a surface layer of this molybdate protects the electrode material from the attack of HF in the electrolyte and facilitates electron transfer. Electroconducting nanolayers of Na_2_MoO_4_ autogenously form on the surface of Na_0.44_MnO_2_ particles, driving superior electrode performance [42]. These surface layers gradually transformed into MoF_6_ and MoO_2_F_2_ layers in the presence of HF.

It is worth noting that part of the sodium atoms can vaporize during the ceramic synthesis of the sodium manganates, and an excess of sodium (typically 5%) is employed in many syntheses. Thus, it is not easy to control the final Na/Mn ratio and stoichiometry [43]. According to recent DFT calculations, vacancies and defects greatly impact the electrochemical performance of Na_0.44_MnO_2_ [44]. Oxygen vacancies decrease the (de)intercalation voltage, while Mn vacancies increase the voltage, and defects improve sodium diffusivity.

While the compounds Na_x_MnO_2_ with 0.22 ≤ x ≤ 0.44 possess a tunnel-type structure, a mixture between tunneled and layered structures is obtained for 0.66 < x ≤ 1.0. The tunneled compound has a limited capacity, while the layered form has a larger capacity but poorer cycling stability. The layered compound Na_2_Mn_3_O_7_, with a triclinic structure, can deliver higher voltage (up to 4.7 V), and the oxygen redox contributes to an exceptionally large capacity (250 mAh g^−1^) [45,46,47,48,49]. However, the stability of the oxygen redox processes is not particularly good. To enhance the electrochemical behavior, Zheng et al. proposed to employ the Na_0.44_MnO_2_/Na_2_Mn_3_O_7_ heterojunction material [50]. The pillar function of tunnel-type Na_0.44_MnO_2_ improves the coulombic efficiency and cycling stability of Na_2_Mn_3_O_7_. Thus, the heterojunction could be a valid strategy.

Magnesium-doping is another strategy to stabilize the tunnel structure, although too much Mg forms the layered structure. Thus, Na_0.44_Mn_0.95_Mg_0.05_O_2_ exhibits lower voltage polarization and superior long-cycle stability [51]. This may be because the Mg-doping facilitates sodium mobility and increases the electrons near the Femi level. A very new strategy to improve the rate capability and cycling stability is the medium-entropy substitution of tunnel-type sodium manganate—Na_0.44_Mn_0.97_Al_0.01_Ti_0.01_Co_0.01_O_2_ [52].

Tevar et al. reported that the materials made with a solid-state synthesis and Na:Mn precursor ratio equal to 0.55 contained Na_0.44_MnO_2_, as well β-Na_0.70_MnO_2_ and α-Mn_2_O_3_ minor impurity phases [43]. According to Zhang et al., the sodium-rich compound Na_0.6_MnO_2_ can be prepared with a slightly different and novel structure also possessing S-tunnel, and they indexed the XRD pattern to an orthorhombic lattice [53]. This material can be prepared with the aid of the surfactant CTAB, and it delivers higher capacity and superior cycling stability. In contrast, the CTAB-free Na_0.6_MnO_2_ material is a mixture of tunnel and layer structures and it presents less charge capacity due to the lower amount of sodium in Na_0.44_MnO_2_ compared to CTAB-Na_0.6_MnO_2_. The guidance of the CTAB surfactant helps to form the Na-rich particles with rod-shaped morphology.

### 2.4. Tunnel-Type MnO_2_

**Figure 6:**
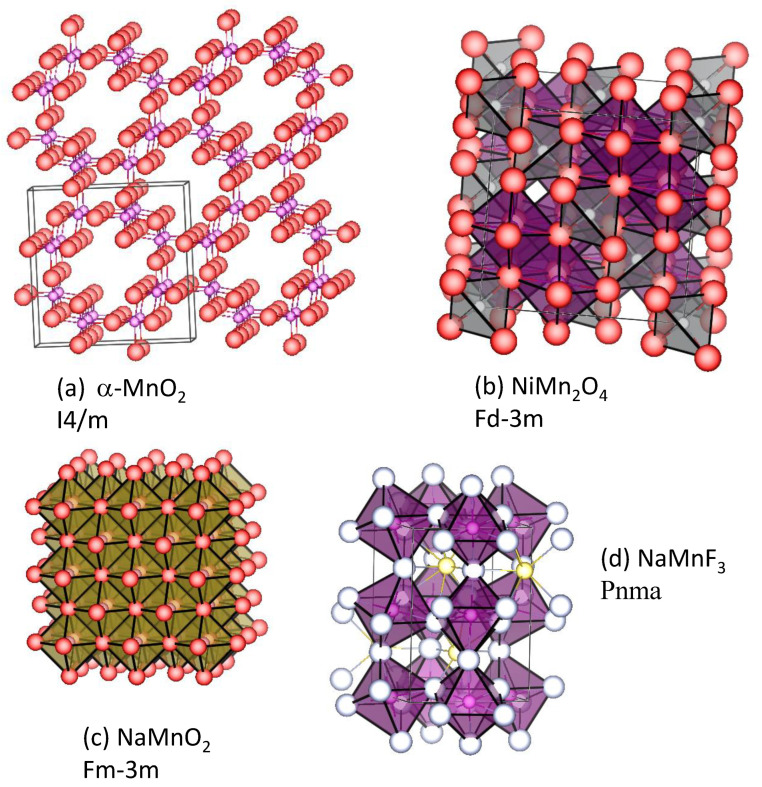
Structures of selected manganese compounds for sodium batteries. (**a**) Tetragonal α-MnO_2_ (I4/m) [57]. (**b**) Cubic spinel NiMn_2_O_4_ [58]. (**c**) Cation-disordered rock salt-type NaMnO_2_ [59]. (**d**) Perovskite-type NaMnF_3_ [60].

The manganese oxide polymorph α-MnO_2_ (hollandite-type) possesses a tetragonal 1D structure (s.g. I4/m) with tunnels (Figure 6), and it can be prepared with nanorods and nanoflowers morphology through hydrothermal method [54,55]. The sodium ions can diffuse easily through the open channels. A higher capacity for NaClO_4_/PC-FEC electrolyte than NaPF_6_/EC-DMC-FEC electrolyte was observable, but the capacity fading is severe in NaClO_4_/PC-FEC and at high current density, and this might be due to the decomposition of PC on the MnO_2_ particle surface and the consequent cell polarization. The additive (typically 5%) fluoroethylene carbonate (FEC) forms a stable SEI film and improves the efficiency and stability of the Na cell, but it introduces polarization. The theoretically calculated insertion voltage is 3.42 [54] or 3.23 V [56], but the experimental voltage range is 1.0–40 V. The calculated diffusion barrier energy is as low as 0.21 eV, and sodium diffusion would then be easy.

Li et al. obtained nanoparticles of α-MnO_2_ with feather-like morphology in situ grown on carbon paper via hydrothermal method [61]. This nanostructured electrode is binder-free. The composite material α-MnO_2_/carbon paper may possess pseudocapacitive behavior, a working voltage between 0 and 3.0 V, and it delivers a maximum reversible capacity of 519 mAh g^−1^ and about. 300 mAh g^−1^ after 400 cycles.

Silver atoms can incorporate into the structure of α-MnO_2_ [62], leading to silver hollandite (Ag_x_Mn_8_O_16_) isostructural to α-MnO_2_ (PDF # 01−077−1987). Silver atoms are in the middle of the tunnels and are electrochemically active. During the discharge process and sodium intercalation into Ag_1.22_Mn_8_O_16_, silver ions reduce to silver metal (cubic Ag) through a reduction-displacement reaction, the charge transfer resistance is decreased, and the crystallinity is reduced for Na_8_Ag_1.22_Mn_8_O_16_. The structure is unstable for a wide voltage window (3.8–1.3 V vs. Na^+^/Na) and a high sodiation level.

The polymorph β-MnO_2_ phase exhibits better electrochemical performance and cyclability than α-MnO_2_ [63]. The tetragonal phase β-MnO_2_ (JCPDS no. 24-0735) possesses a tunnel density of two tunnels per formula unit (0.104 Å^−^^2^), which is more than twice that of α-MnO_2_ [64]. According to the XRD results, the lattice of β-MnO_2_ slightly expands during the reversible sodium intercalation, and a small amount of NaMn_2_O_4_ (s.g. Pnam) forms during the cycling process while the tetragonal phase β-Na_x_MnO_2_ is maintained as the main phase. The nanorods particles of β-MnO_2_ deliver an initial capacity of 350 mAh g^−1^ in sodium cells, and the reversible capacity is about 200 mAh g^−1^ after 100 cycles. The formation of NaMn_2_O_4_ could be the main cause of the gradual capacity deterioration.

### 2.5. Amorphous MnO_2_

Amorphous materials can be an alternative to well-crystallized structures for sodium intercalation. As a result of the amorphous character, the higher concentration of interfacial regions and the small particle size could facilitate rapid sodium diffusion and excellent cyclability. Spherical particles of mostly amorphous MnO_2_ with a 20–60 nm diameter can be prepared by reduction of KMnO_4_ in ethanol [65]. The capacity was maintained at about 137 mAh g^−1^ after 100 cycles, with just a 5% decay of the initial capacity. The charge transfer at the electrode–electrolyte interface improved during the electrochemical cycling of amorphous manganese dioxide. However, a disadvantage is the intrinsic low electronic conductivity at room temperature of MnO_2_, which limits the rate capability.

### 2.6. Spinel-Type NaNi_0.5_Mn_1.5_O_4_

The insertion of sodium into the spinel structure proceeds at a significantly lower voltage than for lithium (typically ca. 1 V lesser). To compensate for that, the redox pair Ni^4+^/Ni^2+^ can increase the average cell voltage of the spinel electrode by about 0.6 V compared to Mn^4+^/Mn^3+^ [10,66]. Sodium intercalation in Ni_0.5_Mn_1.5_O_4_, which is prepared by the delithiation of LiNi_0.5_Mn_1.5_O_4_, occurs exclusively at 8a tetrahedral sites at ca. 3.6 V. Spinel-type NaNi_0.5_Mn_1.5_O_4_ exhibits poorer electrochemical performance in sodium cell than LiNi_0.5_Mn_1.5_O_4_ in lithium cell. The main reason for that can be the larger size of Na^+^ than Li^+^ and the structure distortion induced by the desodiation [67]. The lattice mismatch between sodiated and desodiated spinel results in larger stresses and low cyclability.

Kim et al. theoretically studied the replacement of Mn by Ti in the spinel Na_1−x_[Ni_0.5_Mn_1.375_Ti_0.125_]O_4_ by DFT calculations [68]. The lower electronegativity of Ti could increase the ionicity of the bonding and then help stabilize the crystal structure. The bond Ti-O is stronger than the bond Mn-O. In addition, the larger ionic radii of Ti^4+^ in octahedral coordination (TiO_6_) would widen the diffusion path of Na^+^. The amount of Ti must be limited to avoid the formation of many Jahn–Teller ions Mn^3+^. Nickel would be the only redox center (Ni^4+^/Ni^2+^) during the electrochemical cycling. Ti-doping theoretically mitigates phase segregation, stabilizes the intermediate state, and improves reversibility and cyclability. The positive effect of Ti-doping in LiNi_0.5_Ti_x_Mn_1.5−x_O_4_ was experimentally checked in lithium cells [69], but it seems that Na_1−x_[Ni_0.5_Mn_1.375_Ti_0.125_]O_4_ in sodium cells has not yet been experimentally studied.

### 2.7. Disordered Rock Salt Oxide

Stochiometric NaMnO_2_ with a cation-disordered rock salt-type structure is a metastable polymorph of sodium manganate in which sodium and manganese atoms occur evenly distributed in the same crystallographic site (Figure 6). The reversible capacity (200 mAh g^−1^) in Na cell is higher, the oxidation from Mn^3+^ to Mn^4+^ is highly reversible, and the capacity retention is much better for the nanocrystalline sample obtained by mechanical milling [70]. Na_3_NbO_4_ is non-conductive and electrochemically inactive. The mechanical milling of a mixture of Na_3_NbO_4_ and NaMnO_2_ creates a cationic-disordered rock salt structure in which the Mn^3+^ ions promote electronic transport [70]. The theoretical capacity of Na_1.3_Nb_0.3_Mn_0.4_O_2_ is 311 mAhg^−1^, but the maximum experimental capacity is 200 mAh g^−1^ at 50 °C. During the first charge and extraction of 0.4 Na per formula, Mn oxidizes to Mn^4+^, but during the discharge, manganese reduces to Mn^2+^. Crystallinity diminishes upon electrochemical cycling, and oxygen may evolve during charging. However, niobium is expensive, so Earth-abundant elements such as titanium should replace it. Much better capacity retention was achieved for Nb-free Na_1.14_Mn_0.57_Ti_0.29_O_2_, and this capacity was ascribed to Mn^3+^/Mn^4+^ and anionic O^2−^/O^n−^ redox [71].

### 2.8. Spinel-Type Li_4_Mn_5_O_12_

The cubic spinel Li_4_Mn_5_O_12_ allocates three Li in tetrahedral sites and one in octahedral sites, and it is unstable and decomposes to Li_2_MnO_3_ and LiMn_2_O_4_ at temperatures above 600 °C [72]. Zhang et al. reported that Li_4_Mn_5_O_12_ can reversibly accommodate sodium through a voltage plateau at ca. 2.9 V vs. Na^+^/Na, and with a maximum capacity of ca. 140 mAh g^−1^ (corresponding to 2.2 Na per formula unit) [73]. The capacity is higher for the sample obtained at a lower temperature (400 °C). During charging, Li ions are also removed from the spinel, and Na_x_Li_4−y_Mn_5_O_12_ is formed. The structural changes caused by the sodium-lithium exchange, and the loss of crystallinity upon cycling, originate poorer cycling performance.

### 2.9. Birnessites

Sodium birnessite, Na_x_MnO_2_·yH_2_O, contains crystal water in its layered structure, which may help to reversibly form a metastable spinel-like phase [74]. In contrast to other unwanted and irreversible layered/spinel transformations during alkali ion (de)intercalation, the birnessite can sustain the transformation between the phases. The energy barrier for the migration of the Mn ion from octahedron in the Mn layer to tetrahedron in the Na layer can decrease with the crystal water. Interestingly, the structural water of the birnessite can also be a way to provide high mobility of Mg^2+^ in the oxide framework [75]. The presence of lattice water is strongly associated with sodium storage performance, which is ascribed to the stabilization of the layered structure and the improvement of sodium diffusivity during cycling [76]. Varying the end-of-charge voltage can control the amount of water in the interlayer space. When charged at high voltage, the network water can be withdrawn from the layered structure, simultaneously contributing to a larger reversible capacity and high coulomb efficiency [77].

### 2.10. MgMn_2_O_4_ and Mg_x_Mn_2−y_Fe_y_O_4_

Small guests, such as Li^+^ and Mg^2+^, tend to prefer tetrahedral sites in oxide spinel hosts, while larger Na^+^ ions prefer octahedral sites [14,78]. Cation diffusion in the spinel occurs through successive hops between octahedral and tetrahedral sites, and this fact constrains sodium diffusion. MgMn_2_O_4_ is susceptible to site inversion.

As a new strategy, it has been proposed to firstly remove some magnesium ions from the tetragonal spinel MgMn_2_O_4_, and later to intercalate sodium. [15], for example by oxidation in an electrochemical cell and by chemical disproportionation. The capacity for sodium intercalation in the sample that was not treated with acid is low (31 mAh g^−1^). However, Mg ions can be deintercalated from MgMn_2_O_4_ by disproportionation of Mn(III) in acid solution and dissolution of Mn(III) ions [15], and the resulting compound, Mg_x_Mn_2_O_4_ (x < 1.0), possesses cationic vacancies that can increase the capacity for sodium intercalation. Sodium reversibly intercalates into Mg_0.03_Mn_2_O_4_, delivering a capacity of ca. 100 mAh g^−1^ in the voltage range between 4.1 and 1.9 V. Limiting the charge capacity (or upper cut-off voltage) was critical to achieving good electrochemical cycling, which is in good agreement with the results of Yabuuchi et al. [11].

**Figure 7:**
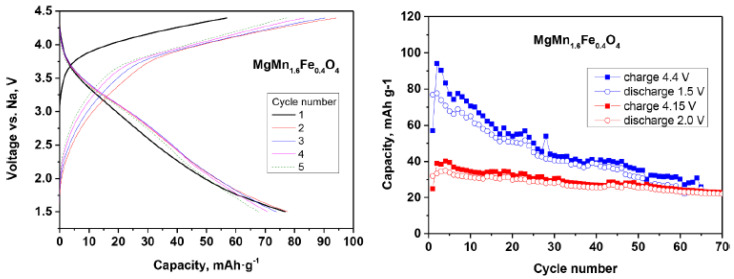
Electrochemical properties of spinel-type MgMn_1.6_Fe_0.4_O_4_ in sodium cell. Reprinted (adapted) with permission from Ref. [15]. License Number 5560700952879 (Elsevier).

Medina et al. explored MgMn_2−y_Fe_y_O_4_ as an electrode for sodium-ion batteries [15]. They proposed that magnesium can help stabilize the structure of the spinel during sodium intercalation, while iron can decrease the irreversible decomposition of the electrolyte solution. The partial replacement of Mn by Fe can decrease the electrolyte decomposition catalyzed by Mn^4+^ on the electrode surface. However, they still only achieved acceptable capacity retention when the upper cut-off voltage was below 4.4 V (Figure 7).

## 3. Sodium Manganese Fluorides and Oxyfluorides

NaM_1−x_Mn_x_F_3_ series with M = Fe, Mn, and Co crystallizes in the orthorhombic space group Pnma with a perovskite-type structure [79] (Figure 6). Although the perovskites generally offer many possibilities for different applications, the atomistic simulations suggest that the energy barrier for sodium diffusion through a three-dimensional path is relatively high in NaM_1−x_Mn_x_F_3_.

Nava-Avendaño et al. studied the fluoro-perovskites Na_2_MnF_5_ (s.g. P2_1_/c), NaMnF_3_ (s.g. Pnma), and unidentified phase of metastable sodium manganese oxyfluoride [80], but the electrochemical (de)insertion of Na in these materials was not practically reached. The strong oxidative decomposition of the electrolyte solution by this fluoride was pointed out. Later, Kitajou et al. reported that the perovskite-type NaMnF_3_, after ball-milling with 20 wt.% of acetylene black, is promising as electrodes for sodium-ion batteries [81]. The theoretically calculated voltage for Na_x_MnF_3_ (0 < x < 1.0) is ca. 4.0 V. The NaMnF_3_/carbon composite mixture has an initial reversible capacity of 89 mAh g^−1^ in the voltage range between 2.0 and 4.3 V, and only 40 mAh g^−1^ after 20 cycles. Thus, it seems that the manganese fluoride can operate at very high voltages, but the capacity retention is not good, most likely due to the decomposition of the electrolyte solution. The theoretical calculations indicated that NaMnF_3_ has three voltage plateaus for the ranges of 0 < x < 0.5 (3.84 V), 0.5 < x < 0.75 (3.9 V), and 0.75 < x < 1 (4.55 V). and the experimental voltage agrees well with the DFT calculations [81].

The sodium insertion into the F-doped spinel Li_1.1_Mn_1.5_Ni_0.5_O_3.8_F_0.2_ was studied by Kim and Amatucci [66]. For that purpose, firstly Li was electrochemically removed from Li_1.1_Mn_1.5_Ni_0.5_O_3.8_F_0.2_, and λ-Mn_1.5_Ni_0.5_O_3.8_F_0.2_ was obtained. The lower voltage of sodium intercalation compared to lithium intercalation (ca. 1.0 V lower) could be partially compensated by the inductive effect of fluorine. The full sodiation of the spinel was achieved, but it was found that the kinetics of the sodium insertion was very limited by the particle size of the spinel.

The oxyfluoride with nominal composition Na_2_MnO_2_F has a disordered rock salt structure where Na and Mn randomly occupy 4a octahedral sites, and O and F occupy 4b sites [82]. The initially observed reversible capacity of ca. 220 mAh g^−1^ at a slow rate corresponds to the desodiation of 1.7 Na per formula and the Mn^3+^/Mn^4+^ redox pair. The capacity is stable during 50 cycles (100 mAh g^−1^ at a high rate). The possible reasons for the observed capacity after further cycles loss can be irreversible anionic redox of oxygen and manganese dissolution.

Anionic doping can strongly modify the crystallographic structure of sodium manganese oxide. Zan et al. reported that after F-doping the tunnel-type Na_0.4_MnO_2_, the resulting compounds with general Na_x_MnO_2−y_F_y_ can be described as an intergrowth of tunnel-type and layer-type (P2) structures, and it exhibits improved cycling stability [83]. As the fluorine content increases, the layer/tunnel ratio increases. The substitution of Mn by Al and O by F in tunnel-type Na_0.4_MnO_2_ results in a P2-type layered structure (s.s. P63/mmc) for the compound Na_0.46_Mn_0.93_Al_0.07_O_1.79_F_0.21_ [84]. For Na_0.66_[Mn_0.66_Ti_0.34_]O_2−x_F_x_ (x < 0.1) with tunnel-type structure, the lattice cell parameters can be tuned by controlling the F-doping [85]. The F-doping enlarges the size of the S-shape tunnels, and this can be related to the electronegativity of fluorine. In addition, F-doping prevents the unit cell change during (de)sodiation and cycling is improved (1000 cycles).

## 4. Polyanion Compounds

**Figure 8:**
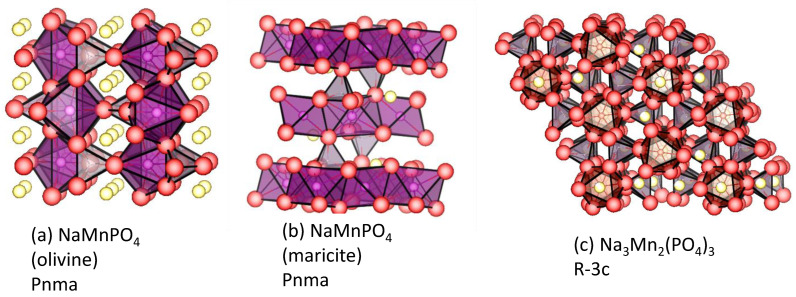
Structures of selected manganese compounds for sodium batteries based on polyanions. (**a**) Maricite-type NaMnPO_4_. (**b**) Olivine-type NaMnPO_4_. (**c**) NASICON-type Na_3_Mn_2_(PO_4_)_3_.

As compared with oxides, polyanion-type active cathode materials offer important advantages in Na-ion batteries. First, the framework of anions, e.g., the structures shown in Figure 8, is commonly very stable and hinder electrode degradation upon cycling. Second, the larger interstices in the structure permit the transport of relatively large sodium ions through the structure. Finally, the strong covalent oxygen bonds with the central atom of the oxoanions impede oxygen evolution, with the subsequent improvement in safety. On the contrary, the electronic conductivity is limited by the anions blocking the contact between the orbitals of the transition metal ions. In addition, the larger molecular weight of the polyanion as compared with an oxide anion may reduce the overall specific capacity. Nevertheless, successful polyanionic materials are nowadays one of the most used cathodes in LIBs.

### 4.1. Maricite and Olivine

An analogous composition to the successful olivine-structure LiFePO_4_ in LIBs is known for sodium and manganese. NaMnPO_4_ crystallizes in two closely related but different structures: olivine (o-NMP) type and maricite (m-NMP) (Figure 8). The framework of both modifications is composed of the phosphate groups, while the metals occupy two sets of equivalent positions commonly referred to as M1 and M2. The structural difference results from the occupancy of these sites: Na^+^ in M1 and Mn^2+^ in M2 for the olivine structure, while the opposite is true for the maricite structure [86]. In the olivine structure, sodium octahedra order in such a way that form zig–zag chains along the b-axis, which ensure a favorable intercalation pathway for alkali ions [87]. Contrary to the olivine structure, the reverse cationic distribution in the maricite structure blocks the pathways for Na^+^ diffusion, thus the maricite is electrochemically inactive [88]. However, the experimental findings demonstrate that the electrochemical performance of m-NMP is boosted by choosing an appropriate synthesis procedure. In this way, Venkatachalam et al. reported the preparation of well-crystallized and pure maricite NaMnPO_4_ nanorods by a polyol procedure [89]. This material displayed interesting electrochemical activity in sodium half-cells, with an initial specific discharge capacity of 102 mAh g^−1^ at 0.1 C [89]. Based on first-principles calculations, it has been demonstrated that the maricite NaMnPO_4_ becomes a semiconductor upon sodium extraction, which occurs in a voltage window between 5.132 V and 4.655 V [90].

Regarding o-NMP, its electrochemical activity was improved by a different approach. Thus, Boyadzhieva et al. [91] used an Mg-doping strategy, which strongly modified the olivine structure by incorporating the doping Mg^2+^ anions in the Na^+^ sites. As a result, reversible capacities close to 100 mAh g^−1^ resulted for sodium half-cells in the 2.1–4.5 V interval. Calculations predict that the doping of o-NMP with Sb causes an enhancement in electronic conductivity and Na diffusion, which is of importance in improving the electrochemical performance of o-NMP [92]. The doping approach seems more effective in respect of the storage properties of o-NMP than the classical approach including the carbon coating of o-NMP [91].

### 4.2. Fluorophosphates

In contrast with the layered structure of Na_2_FePO_4_F, the manganese compound Na_2_MnPO_4_F occurs as a 3D tunnel monoclinic structure belonging to the P21/n space group [93]. Wu et al. first reported a significant capacity (98 mAh g^−1^) in sodium cells [94]. In addition, they prepared different mixed salts in the Na_2_Fe_1−x_Mn_x_PO_4_F and found that for x = 0.3 or higher, the structural transition from 2D to 3D took place. For x = 0.5, Xie et al. reported a capacity of 107 mAh g^−1^ in the 2.0–4.5 V interval [95]. The electrochemical activity of Na_2_MnPO_4_F was boosted by decreasing particle size and carbon coating, which led to an initial discharge capacity of 98 mAh g^−1^. More recently, a specific capacity of 120 mAh g^−1^ was reported for Na_2_MnPO_4_F obtained by a sol-gel route with a citric acid precursor of the carbon coating. The observed cell potential was between the values obtained by first-principles calculations for the following reactions [96]:

Na_2_MnPO_4_F = NaMnPO_4_F + Na  3.7 V (1)

NaMnPO_4_F = MnPO_4_F + Na    4.7 V (2)

### 4.3. Carbonophosphates

**Figure 9:**
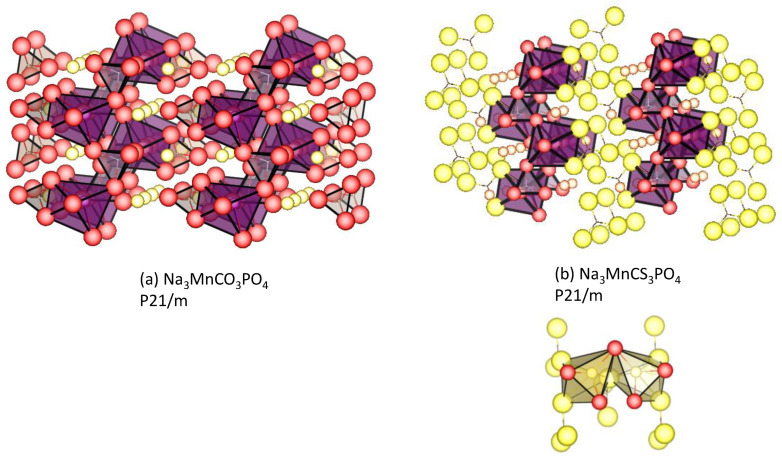
Structures of (**a**) Na_3_MnPO_4_CO_3_ and (**b**) Na_3_MnPO_4_CS_3_ [102].

In general, sodium manganese phosphates display worse electrochemical performance than sodium iron phosphates. To take advantage of manganese-based polyanionic compounds over iron analogues, sodium manganese carbonophosphates emerge as a new family of electrodes for SIB. The first member of this family is sidorenkite—Na_3_MnPO_4_CO_3_ [97,98,99]. The structure consists of double layers built from MnO_6_ octahedra and PO_4_ tetrahedra (Figure 9). The CO_3_ groups are located between double layers to ensure two different crystal positions for Na atoms. From the structure of Na_3_MnPO_4_CO_3_, two Na per formula unit can be extracted thanks to the electrochemical activity of Mn^2+^/Mn^3+^ and Mn^3+^/Mn^4+^ redox couples, with the theoretical capacity being 191 mAh g^−1^. Because of the low electronic conductivity, the electrochemical performance of carbonophosphates depends critically on the method of synthesis and electrode fabrication. Using the hydrothermal method followed by high-energy ball milling with carbon additives, Wang et al. succeeded in the preparation of nano-sized Na_3_MnCO_3_PO_4_, which displays the enhanced electronic conductivity and specific capacity reaching 92.5% of its theoretical one [100,101].

**Figure 10:**
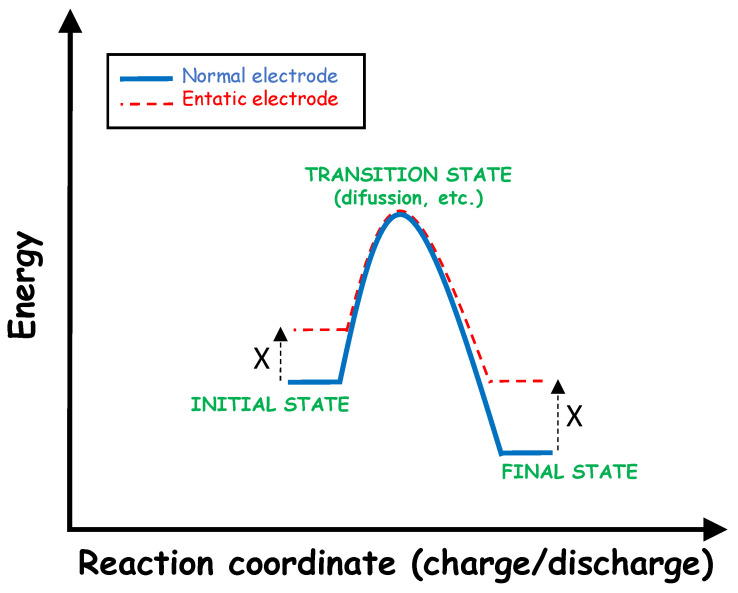
Schematic diagram for a one-step process of two types of electrode materials [102]. Continue blue line: normal process. Dotted red line: entatic state path. The strain energy is X.

According to new DFT calculations on carbonophosphates, the replacement of O by S expands the lattice, and the calculated voltage for sodium extraction from Na_3_MnPO_4_CS_3_ is lower as compared to Na_3_MnPO_4_CO_3_ [102]. Both oxygen (harder base) and sulphur (softer base) atoms surround each sodium atom in a distorted polyhedron (Figure 9). This type of coordination of a metallic element (Mn) surrounded by distinct types of anions (PO_4_^3−^, CO_3_^2−^, and CS_3_^2−^) resembles an energized or entatic state. In the entatic state of the metalloproteins, the protein that surrounds the metal ion imposes a distorted geometry to the coordination sphere of the metal-ligand complex, increasing the energy state of the metal and decreasing the activation energy for certain reactions. The coordination sphere which forms with ligands of different natures can increase the energy of the electrode material and decrease the activation energy for the charge/discharge process (Figure 10). The concept of an entatic state is so attractive that it extends to enzymes, organic chemistry, catalysis, metallorganic frameworks, and even electrode materials [102]. For example, the distortion of the unit cell due to the Jahn–Teller effect of Mn(III) in the spinels can be cancelled by this multianion approach [102], and the charge/discharge process may be more efficient.

### 4.4. NASICON-Related Compounds

A thoroughly studied polyanionic cathode material is Na_3_V_2_(PO_4_)_3_ (NVP) with a NASICON-related structure (Figure 8). It generates from “lantern” units of three PO_4_ tetrahedra sharing corners with two VO_6_ octahedra. These units link to others defining different sets of equivalent positions available to sodium. The resulting material has high ionic conductivity but poor electronic conductivity, thus needing composites with conductive carbon. NVP in sodium half-cells displays electrochemical activity in a wide range of working potentials (0.3–4.7 V) with different plateaus. The most common cathodic activity involves a 3.6 V plateau in which two Na can be reversibly extracted from the initial NVP stoichiometry. A second plateau around 4.6 V could extract the third sodium.

The most common preparative route reported for the NASICON phosphates is the citric-based sol-gel method. This procedure generates a reducing atmosphere during the calcination step in inert atmosphere. It allows both to preserve a low valence state in the transition metals and coat the active material with a conductive carbon phase. Notwithstanding, alternative synthetic procedures have allowed researchers to design new morphologies [103], 3D pore structures [104], and new carbon composites [105] to optimize their electrochemical behavior. The replacement of vanadium by manganese could provide extra value to this system because of its greater abundance and being more environmentally friendly. First-principles calculations on Na_x_Mn_2_(PO_4_)_3_ revealed low favorable formation energies [106]. The possible participation of different redox pairs Mn^3+^/Mn^2+^ and Mn^4+^/Mn^3+^ was also shown. However, the Jahn–Teller distortion of the Mn^3+^ ions impedes its use as the only transition metal in the structure. For these reasons, manganese usually appears combined with other metals. Zhou reported Na_4_MnV(PO_4_)_3_ in which Mn^3+^/Mn^2+^ and V^4+^/V^3+^ redox couples are respectively accessed at 3.6 and 3.3 V, delivering an initial efficiency as high as 97% and long cycling durability at 10 C [107]. This compound can be prepared in a fibrous particle morphology by using a surfactant-assisted method. According to XPS data, the presence of V^3+^, Mn^2+^, and Mn^3+^ accounted for the sodium-rich stoichiometry. The material displayed an interesting specific capacity of 58 mAh g^−1^ when cycled at 5 A g^−1^ and a capacity retention of 85.1% over 1200 cycles at 1 A g^−1^ [108]. In addition, two pairs of apparent plateaus at around 3.4 V and 3.6 V vs. Na^+^/Na are ascribed to the V^3+^/V^4+^ and Mn^2+^/Mn^3+^ redox pairs, respectively.

Zirconium was also combined in 1:1 proportion with manganese in Na_3_MnZr(PO_4_)_3_ and the nanometric particles prepared by Gao et al. [109]. The authors suggested that although the Mn^3+^O_6_ octahedra are distorted on the local scale, the cooperative Jahn–Teller distortion that would result in a long-range ordering of the Na ions is suppressed in this cathode material. In consequence, the material exhibited excellent cycling stability and 91% capacity retention after 500 cycles at 0.5 C rate. Na_3_MnZr(PO_4_)_3_ co-functionalized with semi-graphitic carbon and reduced graphene oxide was evaluated by Zhu et al. [110]. When used as the cathode in sodium half cells, it provided a reversible capacity of 114 mAh g^−1^ at 0.2 C. The dual carbon functionalized material was also evaluated in full SIB vs. soft carbon, which delivered an initial discharge capacity of 97 mAh g^−1^ and 73% after 100 cycles at 0.2 C. However, the initial coulombic efficiency was low but increased upon cycling. More recently, Ma et al. achieved excellent performance at 50 C and long-term cycling stability in a wide temperature range by preparing a composite of reduced graphene oxide and amorphous carbon in Na_3_MnZr(PO_4_)_3_ microspheres. The resulting interconnected conductive network provided high porosity and specific surface area, allowing to accommodate the volume changes, and achieving fast sodium storage by ameliorating the electrode–electrolyte interface [111].

**Figure 11:**
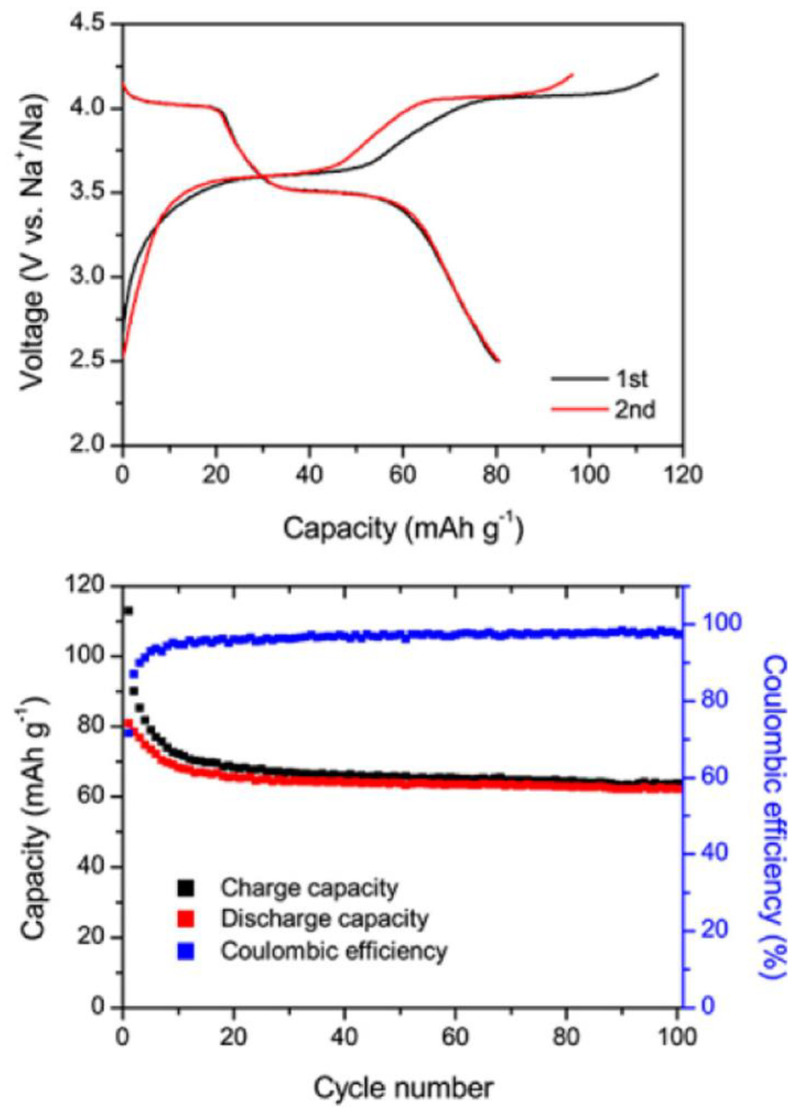
Electrochemical properties of NASICON-type Na_3_MnTi(PO_4_)_3_ in sodium cell. Reprinted (adapted) with permission from Ref. [112]. Copyright (2016) ACS.

The combination of manganese with titanium in Na_3_MnTi(PO_4_)_3_ has also attracted more attention due to the eco-friendly and low-cost benefits of these elements. Gao et al. first reported this composition as a structurally stable framework able to deliver two sodium ions per formula unit through access to both Mn^3+^/Mn^2+^ and Mn^4+^/Mn^3+^ redox couples (Figure 11). The minimum voltage gap of 0.5 V between these plateaus favors the applicability of this electrode material as a high-voltage cathode for sodium-ion batteries [112,113]. Further reports have even remarked the possibility of assembling symmetric full cells with Na_3_MnTi(PO_4_)_3_ as both positive and negative because of the significant differences in the potential of the distinct redox couples of Mn^3+/4+^ (ca. 4 V) and Ti^3+/4+^ (ca. 2 V) [113]. Notwithstanding, its poor electronic conductivity requires carbon coating to achieve adequate rates of electron transfer. Different authors have proposed interesting strategies to prepare highly conductive carbon composites. Thus, Li et al. reported graphene-encapsulated Na_3_MnTi(PO_4_)_3_ particles with carbon-shell covering material. The synergistic effect of this multifunctional 3D conductive network enhances the contribution of pseudocapacitance to eventually provide outstanding rate capability and cycling stability [114]. Otherwise, Na_3_MnTi(PO_4_)_3_ particles embedded in a nitrogen-doped carbon matrix are evidenced to be an interesting solution to enhance the electronic conduction of this NASICON material, resulting in a notorious improvement of the specific capacity and high-rate capability [115].

The incorporation of chromium in Na_4_MnCr(PO_4_)_3_ has also been proposed as an interesting three-electron reaction, involving Mn^2+^/Mn^3+^, Mn^3+^/Mn^4+^, and Cr^3+^/Cr^4+^ redox couples, which can deliver a high energy density (566.5 W h kg^−1^) for this kind of phosphate Na-storage cathode material [116]. Alternatively, the partial replacement of manganese and vanadium by this transition metal has led to a significant improvement in sodium diffusivity along with a net gain of the reversible extraction of sodium at either low or high rates [117,118]. Eventually, Zheng et al. synthesized the aluminum-containing compound by a sol-gel method. Despite the economic and environmental benefits and higher energy density as compared to vanadium compound, Na_3_MnAl(PO_4_)_3_ exhibited low capacity and undesirable cycling stability [119].

### 4.5. Silicates

**Figure 12:**
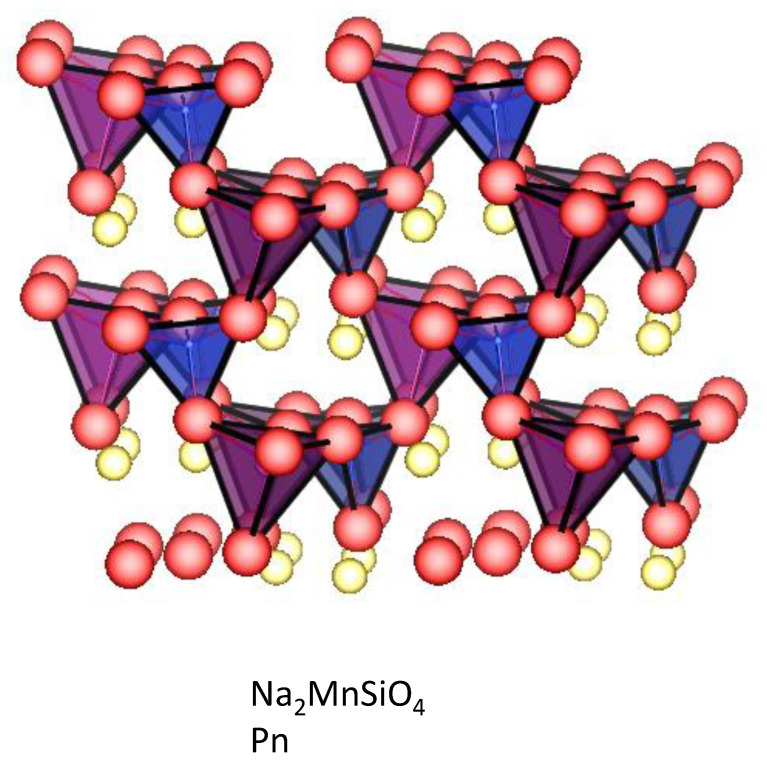
Structure of Na_2_MnSiO_4_.

As in the case of carbonophosphates, sodium manganese silicates can intercalate Na^+^ ions through a two-electron reaction due to the redox couples Mn^2+^/Mn^3+^/Mn^4+^ [120]. This allows us to achieve extremely high capacities. The structure of polyanionic Na_2_MnS_i_O_4_ is depicted in Figure 12. The monoclinic Na_2_MnSiO_4_ covered with an amorphous carbon film with a thickness of around 2–3 nm delivers the highest specific capacity (i.e., 210 mAh g^−1^) in comparison with other polyanionic compounds [121]. The excellent electrochemical properties of Na_2_MnSiO_4_ are a result of the energetically favorable diffusion of Na+, which is faster than Li^+^ diffusion into Li_2_MnSiO_4_ [122]. The Na ion diffusion takes place by 3D path with the migration energy of 0.81 eV [123]. Further improvement of the electrochemical properties of Na_2_MnSiO_4_ through selective doping with aliovalent Al^3+^ ions needs to be proven experimentally [123].

## 5. Prussian Blue Analogues

**Figure 13:**
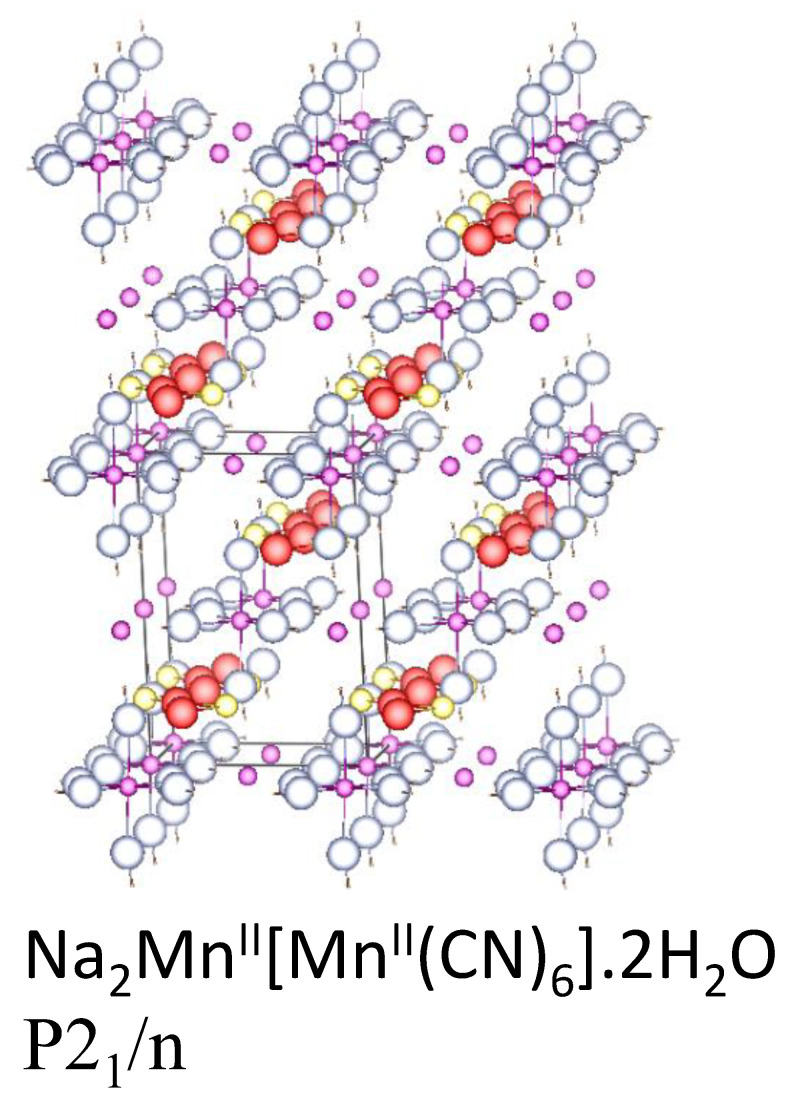
Structure of Na_2_Mn^II^[Mn^II^(CN)_6_]·2H_2_O [126].

Goodenough’s group pointed out that the bond between oxygen and sodium can impede its motion while the replacement of O^2−^ by CN^−^ decreases the activation energy for sodium transfer and, thus, sodium manganese hexacyanoferrate is attractive as a cathode [124]. Metal hexacyanoferrates with general formula A_x_M[Fe(CN)_6_]_y_·zH_2_O, where A = Li, Na, K, etc., M = Fe, Mn, Co, Ni, Cu, etc., 0 < x < 2, and 0 < y < 1, are known as Prussian Blue analogues (PBAs) [125]. Sodium ions easily accommodate in the spacious channels of the PBAs, which typically possess a face-centered cubic unit cell (s.g. Fm-3m). This structure provides rapid sodium mobility. The crystal structure of sodium manganese hexacyanomanganate (Na_x_Mn[Mn(CN)_6_]) depends on the sodium content and oxidation state of manganese. Na_2_Mn^II^[Mn^II^(CN)_6_]·2H_2_O possesses a monoclinic structure (s.g. P21/n), which is atypical for PBAs (Figure 13) [126]. After oxidation of all the Mn to Mn(III), the structure of Mn^II^[Mn^II^(CN)_6_] is the typical cubic perovskite (Fm-3m), while the structure of the fully reduced electrode (Na_3_Mn^II^[Mn^I^(CN)_6_]) is monoclinic (P2_1_). The capacity Na_2_Mn[Mn(CN)_6_ is very high (209 mAh g^−1^ at 40 mA g^−1^) [127]. Hurlbutt et al. found by DFT that Na_3_Mn^II^[Mn^I^(CN)_6_] and the hydrated form Na_3_Mn^II^[Mn^I^(CN)_6_]·2H_2_O are thermodynamically stable, despite including the rare Mn(I) [128].

It seems that the vacancies of Fe(CN)_6_, the water molecules, and the structure disturbances caused by the redox reactions can deteriorate the life cycle of PBAs. To improve the cycling stability, it has been proposed to employ Na_2_Ni_x_Mn_y_Fe(CN)_6_, where nickel is electrochemically inactive [129]. The compositional disorder would affect the electrochemical reaction. Using a high-entropy material, the phase transition and structural degradation could be suppressed [130]. The DFT calculations unveiled that the structure of the high-entropy PAB is more robust, and the partial replacement of Mn by Ni could contribute to this disorder. Creating a Ce-rich shell on the PBAs particles could improve the stability of the electrode–electrolyte interface [131]. A copper-containing coating layer obtained by a simple exchange method is another option [132].

## 6. Conversion-Type Electrodes

As early as 2000, Jean Marie Tarascon group first reported the use of binary transition metal oxides as anode material for lithium-ion batteries [133]. In this concept, metal reduction to the metallic state together with the formation of lithium oxide were found to occur together with particle comminution and pseudocapacitive phenomena on the deep discharge of lithium test batteries. The process was partially reversible with the regeneration of transition metal oxides with a highly dispersed nature upon cell charge. Poizot et al. also anticipated the difficulties in reducing MnO to Mn metal by Li [134].

The conversion anode concept was further extended to mixed transition metal oxides [135,136,137,138,139] and oxalates [140,141,142]. For example, low-cost Ni_6_MnO_8_ (s.g. Fm3m) material was proposed as anode for lithium-ion batteries in 2002, with an experimental reversible capacity of ca. 700 mAh g^−1^, and the proposed discharge-charge mechanism involved the following reactions [136,143]:

Ni_6_MnO_8_ + (12 + x) Li = 6Ni + 6Li_2_O + Li_x_MnO_2_ (3)

NiO + 2Li = Ni + Li_2_O (4)

Later studies found that metallic Mn can be formed when MnO_x_ oxides are discharged down to 0.0 V vs. Li^+^/Li [144,145]. Another conversion electrode material based on Mn oxide for lithium batteries reported in 2002 was MnCo_2_O_4_, but it exhibits high irreversibility and low coulombic efficiency in the first cycle [146].

In 2002, we also reported the first conversion electrodes to the anode of sodium-ion batteries, using the spinel NiCo_2_O_4_ in the form of nanoparticles obtained by the calcination of oxalates. A full Na-ion cell was also studied, using Na_x_CoO_2_ as the cathode material [137]. This possibility was developed in further work by extending the concept to other oxides and oxysalts [147,148,149]. The general equation to describe the conversion process in oxides is:

MO_x_ + 2xe^−^ +2xNa^+^ → M + xNa_2_O (5)

where M represents single or multiple transition metals with an average oxidation state of +2x. In addition to this conversion-type reaction, it is generally accepted that the pseudocapacitive behavior also contributes to the capacity of these electrodes. A main drawback is that the volume change of the electrode material during the charge-discharge is even larger for sodium than lithium.

Although it is generally accepted that Fe, Co, Ni, Cu, and Nb oxides are particularly attractive as potential conversion anodes in Na-ion batteries [150], recent reports have examined the possible extension to manganese oxides. This section is focused on the electrodes based on manganese compounds, which would be used as conversion anodes in sodium-ion batteries and involve reduction down to ca. 0 V vs. Na^+^/Na.

### 6.1. Manganese Oxides

To achieve good electrochemical performance, the particles of MnO are prepared with special morphologies and in the form of MnO-carbon composites. Cauliflower-like MnO-carbon composite materials prepared by a hydrothermal method have been studied in both sodium and lithium half-cells. Although the capacities and capacity retention were superior in lithium cells, the material displayed 123 mAh g^−1^ with good retention up to 200 cycles [151]. The composite formed by ultrasmall MnO nanoparticles (ca. 4 nm of diameter) supported on N-doped carbon nanotubes displays exceptional rate capability (709 mAh g^−1^ at 0.1 A g^−1^) and ultralong cycling life (273 mAh g^−1^ after 3000 cycles) [152]. During the initial discharge, manganese oxide particles anchored on carbon nanotubes are reduced through a conversion reaction:

MnO + 2Na^+^ + 2e^−^ → Mn + Na_2_O (6)

During the subsequent charge, manganese is reversibly oxidized:

Mn + Na_2_O → MnO + 2Na^+^ + 2e^−^ (7)

The carbon nanotubes enhance the electronic conductivity and improve the stability of the electrode.

Sun et al. have questioned the use of MnO as a conversion electrode for SIB because the thermodynamic driving force for sodium storage is lower compared to lithium [153], and the change of the Gibbs energy for conversion reaction (6) is ΔG = −13.5 kJ mol^−1^, while for lithium it is more exergonic:

2Li + MnO = Li_2_O + Mn, ΔG = −198.3 kJ mol^−1^ (8)

Jiang et al. [154] studied different transition metal oxides as potential conversion-type anodes for SIBs. Among them, Mn_3_O_4_ thin films were found to deliver a first discharge capacity of 257 mAhg^−1^ and retained 61% of the initial charge capacity after 200 cycles. The electrochemical performance of Mn_3_O_4_ was found to be better than those of Co_3_O_4_ and NiO, but it was still very satisfactory [154]. Later, the mechanism of the reaction was explored in Mn_3_O_4_ nanoparticles (15–45 nm) without additional carbon (no carbon coating and no carbon template) [155]. Firstly, Mn_3_O_4_ is reduced to MnO together with the formation of Na_2_O at ca. 0.6 V, and then to Mn at ca. 0.4 V. During the charge, metallic Mn is reoxidized to MnO at 0.1 V, and to Mn_3_O_4_ at ca. 0.8 V. The cycling stability is reasonable (158 mAh g^−1^ after 200 cycles).

A microstructure of cubic-like Mn_2_O_3_ with nanoparticles (sub-units) embedded on its porous surface was obtained. The electrochemical results indicate that the Mn_2_O_3_ electrode can deliver a promising discharge capacity, cyclability, and rate capability during the (de)insertion of Na-ions. The Mn_2_O_3_ electrode exhibited a high initial discharge capacity of 544 mAh g^−1^ at 100 mA g^−1^ and retained 130 mAh g^−1^ after 200 cycles [156].

The crystal structure of MnOOH contains tunnels such as β-MnO_2_. This manganese oxyhydroxide in the form of nanorods was reported by Shao et al., who surprisingly suggested that MnOOH nanorods undergo a partial conversion to MnO and NaOH in sodium half-cells down to near 0 V [157]:

MnOOH + Na^+^ + e^−^ → MnO + NaOH (9)

During the charging process, MnOOH is regenerated. The reaction allowed a capacity of 421 mAh g^−1^ at 80 mA g^−1^ with 86.7% coulombic efficiency, which is most likely indicative of irreversible processes while the specific capacity was reduced to 162 mAh g^−1^ at 2000 mA g^−1^.

### 6.2. Manganese-Based Spinels and Perovskites

The spinels AMn_2_O_4_ (A = Co) and MnA_2_O_4_ (A = Fe) have attracted little attention as conversion electrodes for SIB. A main problem for these electrode materials is that the conversion reaction during the first discharge irreversibly consumes sodium, and the initial coulombic efficiency is low.

**Figure 14:**
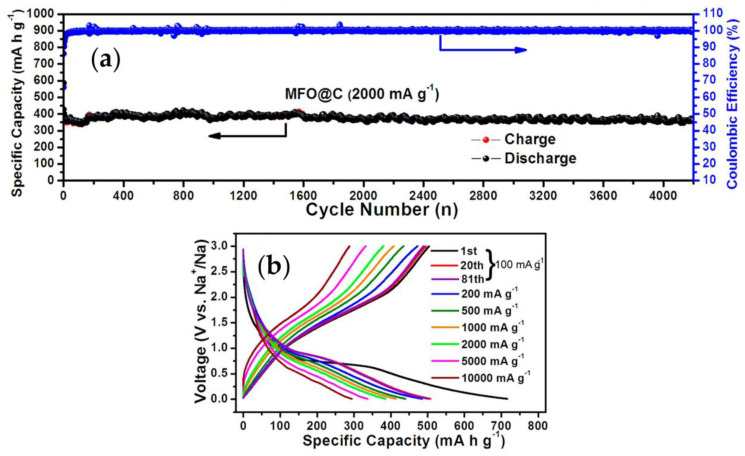
Electrochemical properties of MnFe_2_O_4_@C in sodium cell. Reprinted (adapted) with permission from Ref. [159]. Copyright (2016) ACS.

The composite MnFe_2_O_4_/reduced graphene oxide delivers a stable capacity of 258 mAh g^−1^ at 0.1 C rate for 50 cycles in sodium cells [158]. Fe, Mn, and Na_2_O are formed at 0 V through a conversion reaction, while Fe_2_O_3_ and MnO are detected at the charge state (3.0 V). The graphene sheets enhance the electronic conductivity of the composite, buffer the volume expansion during the conversion reaction, and act as a barrier to avoid active material dissolution. The Na-alginate binder provides strong active material-current collector interaction. Binder-free nanodots of MnFe_2_O_4_ encapsulated in carbon nanofibers, and prepared via the electrospinning method, have particularly good rate-capability and an ultralong cycling life (Figure 14) [159].

Yuan et al. used the electrostatic spray deposition technique to fabricate porous CoMn_2_O_4_ on Ni foam, which was proposed as a binder-free electrode [160]. The mechanism for sodium storage is like the lithium conversion mechanism, involving the formation of Co, Mn, and Na_2_O at ca. 0 V. The reversible capacity is 185 mAh g^−1^ after 50 cycles at 100 mA g^−1^.

Fluorides containing Mn and with perovskite-type structures are also under investigation. After introducing K^+^ and F^−^ vacancies in the perovskite-type KMnF_3_ (s.g. Pm-3m) and the addition of reduced graphene oxide (rGO), the composite K_0.86_MnF_2.69_@rGO is obtained [161]. Expectedly, the vacancies improve the capacity for sodium intercalation. At about 0.21 V, the fluoride compound is irreversibly converted to Mn metal, KF, and NaF, and the reversible capacity of the composite is only about 40 mAh g^−1^. The electrochemistry of composites perovskite/reduced graphene oxide electrodes is between sodium-ion batteries and capacitors. The compound K_0.71_Ni_0.12_Co_0.41_Mn_0.47_F_2.77_ is vacancy defective [162] and, besides the capacity resulting from cationic conversion/intercalation, the anions (e.g., PF_6_^−^) in the electrolyte can also be reversibly embedded in the electrode. Yang et al. have very recently reported the perovskite fluoride with nominal composition K_0.97_Ni_0.31_Zn_0.28_Mn_0.41_F_2._ [163], and the sodium storage process in this material is based on conversion and alloying (Na-Zn).

## 7. Methods of Synthesis

Although the one-step solid-state method is often employed for the preparation of many compounds, and these methods are ideal for industrial production, more sophisticated methods are more convenient for fine control of the resulting phases and particle morphology. The preparation of the manganese compounds described above could involve the optimization of the methods of synthesis, particularly for nanostructured materials. The most significant advances in this field are reviewed below. The simplest method to reduce the particle size is ball-milling, but the particles obtained usually have irregular morphologies.

The synthesis based on the sol-gel method and precursor method can be particularly useful for the preparation of nanostructured oxides. New nanosized spinels Mg_x_Mn_2−y_Fe_y_O_4_ (0 ≤ y ≤ 2) were prepared through a modification of the Pechini process [15], firstly dissolving magnesium, manganese, and iron nitrates in water, then adding citric acid and ethylene glycol, and final heating of the resulting precursor. The sol-gel method can be very useful for the cationic substitution in NASICON-type cathodes [164]. Together with citrate precursors, for the synthesis of tunnel-type Na_x_MnO_2_, the cetyltrimethylammonium bromide (CTAB) surfactant provides a pathway to tailor the particle morphology [53].

Manganese hexacyanomanganate, Na_2_Mn^II^[Mn^II^(CN)_6_], is typically prepared by co-precipitation method, after adding NaCN to an aqueous solution of manganese nitrate in the presence of excess NaCl, and under N_2_ atmosphere to avoid oxidation of manganese [127,165]. A much more sophisticated method was employed for the anchoring of Na_x_K_y_MnFe(CN)_6_ on hierarchical porous ultrathin carbon networks [166]. This method involves many steps, including the preparation of a template, freeze-drying, and chemical co-precipitation.

The preparation of hollow nanostructures of sodium-manganese compounds for SIB remains little explored, although the recent advances in the preparation of other compounds could be a source of inspiration, for example, the methods based on micelles, solvothermal, and spray-drying [167]. The strategies for the preparation of heterostructures comprise solvothermal/hydrothermal processes, quenching, and other methods [168]. Unfortunately, the methods of preparation of the heterostructures lack the precise control of many properties, such as the interface between the two structures.

## 8. Conclusions and Perspectives

**Figure 15:**
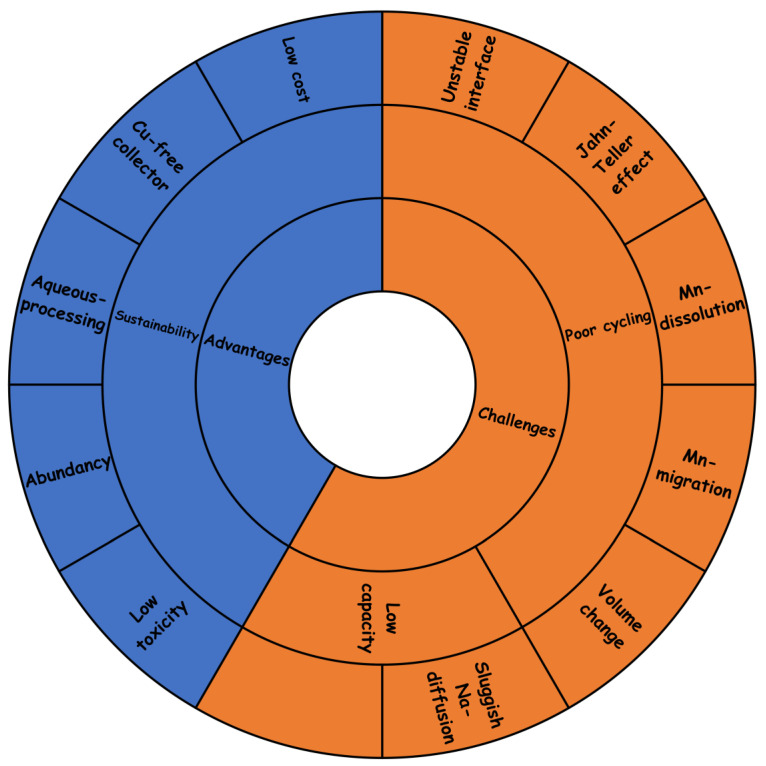
Advantages and challenges of the SIB batteries with manganese compounds.

As a schematic summary, the main advantages and drawbacks are given in Figure 15. Thus, electrode materials made of low-cost elements, such as sodium Na and Mn, are particularly interesting for developing new and sustainable batteries based on green chemistry. Manganese is less toxic than cobalt and nickel. Replacement of lithium by sodium led to avoiding the use of copper in the current collector. These materials could also be adequate for aqueous processing, avoiding organic solvents.

The main drawback of these insertion-electrode materials is their cycling stability, and the Jahn–Teller effect of manganese is a main contributor to the lack of structural stability. Manganese dissolution has been observed for some electrodes. Compared to layered oxides, the tailoring of the composition to control the structural change during electrochemical cycling has been scarcely explored in non-layered oxides. Spinel-type NaMn_2_O_4_ and tunnel-type Na_0.44_MnO_2_ show promise as an efficient and sustainable electrode for SIB, but this material should be tailored to achieve good stability. The possible migration of manganese ions, particularly in the spinel-type structure, could be another source of structural instability. Another main drawback of several of these cathodes, such as the tunnel-type and the spinel-type structure, is the relatively low capacity, particularly at a high rate.

**Figure 16:**
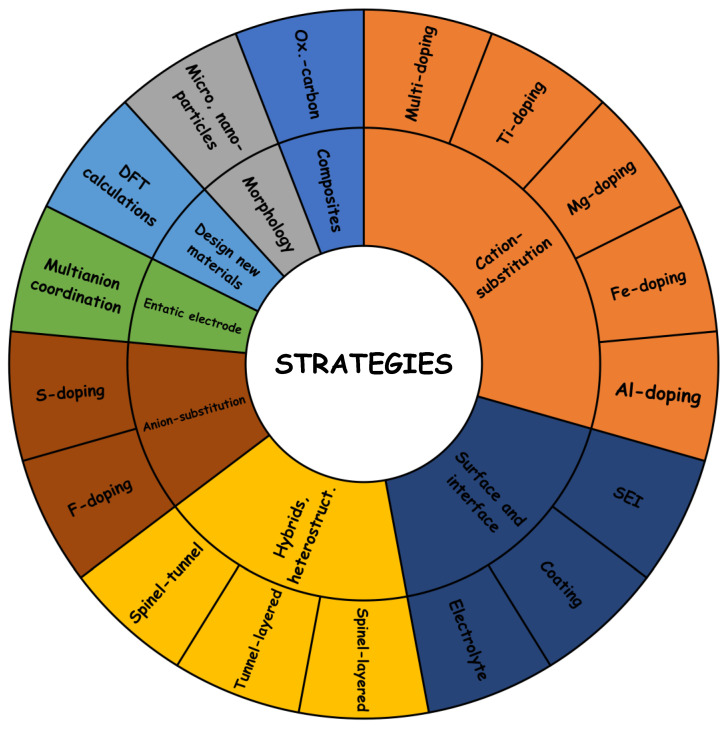
Strategies for improvement of the SIB batteries with manganese compounds.

The main strategies to improve the cyclability and increase the energy density of the manganese oxides can be briefly described below and in Figure 16.

- Building biphasic heterostructures, for example, spinel/layered and tunnel/layered. A synergistic effect could be found for the suitable combinations of two different structures in the same electrode material. For example, the formation of heterostructures or composites could be a strategy to raise the capacity of tunnel-type materials [169];
- Partially replacing atoms (cations and/or anions) or doping to improve structural stability. The large volume expansion/contraction and structure transformation should be avoided, for example suppressing the Jahn–Teller distortion. The partial replacement of the Jahn–Teller ion manganese by non-Jahn–Teller ion can allow to achieve better cycling stability;
- Multianion approach and formation of entatic electrode. For example, partial replacement of O by another anion (fluoride, sulfide, or others) [102]. The coordination of an atom (e.g., sodium and manganese) by different types of anions (e.g., F^−^/O^2−^, S^2−^/O^2−^ and PO_4_^3−^/F^−^) can exert several beneficial effects. In addition, this sort of anion doping can modify the stoichiometry of the compound (Na/Mn ratio) and raise the capacity;
- Replacing Mn by Ni to rise the operating voltage. The redox pair Ni^4+^/Ni^2+^ can provide higher voltage than Mn^4+^/Mn^3+^, with the consequent higher energy density;
- Implementing theoretical calculations to design the materials with thermodynamic stability and optimized properties. DFT calculations are the main tool for selecting new materials, particularly for future materials based on several types of anions;
- Tailoring the particle morphology for shortening the sodium diffusion pathway, particularly in the crystallographic directions in which the mobility of sodium is low. For example, rod morphology is very promising.
- New additives and coatings for the improvement of the electrolyte and interface properties. This involves not only the SEI formation, but also the improvement of cycling life, thermal stability, and safety [170]. Beyond the typical fluoroethylene carbonate (FEC) additive, and formation of NaF-rich SEI in the anode, the additive for SIB should also improve the electrochemistry of the high-voltage cathode (CEI), but this is still in an initial state of exploration compared to LIB.

Regarding new perspectives, we believe that since the perovskite structure offers many possibilities, there is a chance for tailoring the composition and structure of the Mn-containing perovskites for sodium (de)intercalation, such as lithium in Li_0.5_La_0.5_TiO_3_ [171]. The use of pure manganese oxides as conversion electrodes to replace hard-carbon as negative electrode for SIB is doubtful, due to the difficulties in achieving high coulombic efficiency and extended cycling. However, the composites based on manganese oxides and carbon can (e.g., MnFe_2_O_4_@C) exhibit excellent cycling stability [159].

The two more promising strategies for future progress are most likely the multianion approach and the improvement of the SEI. The multianion approach could mitigate the changes of the unit cell during charge/discharge, particularly due to the Jahn–Teller effect, and it could improve the mobility of sodium in the host material. On the other hand, the engineering of the interfaces by coating treatment could contribute to improving the SEI stability and cycling stability. Beyond carbon coating, new types of materials for the coating of the electrode should be explored, such as borates.

Manthiram group recently found that the electrochemical performance of the spinel LiMn_2−x_Fe_x_O_4_ can be greatly improved for materials prepared under oxidative synthesis conditions [172]. The oxygen flow during the synthesis minimizes the oxygen vacancies in the structure of the resulting spinel, and it decreases the surface of the particles. We believe that this strategy should also be explored in sodium-manganese spinels.

A drawback of several of the most significant methods of synthesis is that these are time-consuming and inefficient. It would be necessary to develop new methods of synthesis based on fast procedures and low-energy consumption. For example, ultrafast high-temperature sintering with Joule heating is a promising method that has been applied to NASICON-type solid electrolytes, but it may also be a source of inspiration for electrode materials [173].

## Figures and Tables

**Table 1:** Summary of the relevant Mn-based materials with the non-layered structure for sodium intercalation. Voltage range and capacity values are taken from the experimental results reported in the references.

Electrode Material (Structure Type)	Space Group	Voltage Range, V	Capacity, mAh g^−1^	Refs.
λ-MnO_2_ (spinel)	Fd-3m	2.0–4.0	180	[6]
Mn_2.2_Co_0.27_O_4_ (tetragonal spinel)	I4_1_/amd	1.5–4.0 V	95	[7]
LiMn_2_O_4_ (cubic spinel)	Fd-3m	2.0–4.0	190	[11]
Li_1.2_Mn_1.8_O_4_ (cubic spinel)	Fd-3m	2.0–4.0	65	[11]
NaMn_2_O_4_ (CaFe_2_O_4_ post-spinel)	Pnma	1.6–4.8	80	[20]
Na_0.9_MnSnO_4_ (CaFe_2_O_4_ post-spinel)	Pnma	2.0–4.5	30	[21]
NaMn_2_O_4_ (cubic spinel)/Na_x_MnO_2_ (layered)	Fd-3m	2.2–3.6	181	[12]
Li_2−x_MnO_3_ (cubic spinel)	Fd-3m	1.5–4.2	160–200	[13]
Na_0.44_MnO_2_ (tunnel)	Pbam	2.0–4.0	140	[23]
Na_0.44_Mn_1−x_Ti_x_O_2_	Pbam	1.5–3.8	100–110	[29]
Na_0.44_Mn_0.89_Ti_0.11_O_2_	Pbam	2.0–4.0	71–119	[30]
Na_0.61_[Mn_0.27_Fe_0.34_Ti_0.39_]O_2_	Pbam	2.6–4.2	98	[32]
Na_0.44_Mn_0.9925_Co_0.0075_O_2_	Pbam	2.0–4.0	138	[33]
Na_0.44_MnO_2_ (tunnel)/LiMn_2_O_4_ (cubic spinel)	Pbam/Fd3m	2.0–4.0	120	[24]
Na_0.44_MnO_2_ (tunnel)/Na_2_Mn_3_O_7_(layered)	Pmc2_1_	1.5–4.7	145–278	[26]
Na_0.44_Mn_0.97_Al_0.01_Ti_0.01_Co_0.01_O_2_ (tunnel)	Pbam	2.0–4.0	140	[52]
Ni_0.5_Mn_1.5_O_4_ (cubic spinel)	Fd-3m	2.5–4.7	140	[66]
NaMnO_2_ (disordered rocksalt)	Fm-3m	1.2–4.5	200	[59]
Na_1.3_Nb_0.3_Mn_0.4_O_2_ (disordered rocksalt)	Fm-3m	1.0–4.0	150–200	[70]
Na_1.14_Mn_0.57_Ti_0.29_O_2_ (disordered rocksalt)	Fm-3m	1.2–4.5	200	[71]
Na_2_MnO_2_F (disordered rocksalt)	Fm-3m	1.5–4.5	220	[83]
Na_2_MnF_5_	P2_1_/c	1.0–4.7	ca. 0	[81]
NaMnF_3_ (perovskite)	Pnma	2.0–4.3	89	[82]
K_0.97_Ni_0.31_Zn_0.28_Mn_0.41_F_2.84_@rGO (composite of perovskite and graphene)	Pm-3m	0–3	173	[163]
K_0.86_MnF_2.69_@rGO (composite of perovskite and graphene)	Pm-3m	0–3	40	[161]
Li_1.1_Mn_1.5_Ni_0.5_O_3.8_F_0.2_ (cubic spinel)	Fd-3m	2.5–4.7	140	[66]
Li_4_Mn_5_O_12_ (cubic spinel)	Fd-3m	2.3–3.3	140	[73]
Mg_0.3_Mn_2_O_4_ (cubic spinel)	Fd-3m	1.5–4.4	90–105	[15]
Mg_0.8_Mn_1.9_Fe_0.1_O_4_ (tetragonal spinel)	I4_1_/amd	1.5–4.4	70	[15]
α-MnO_2_ (hollandite)	I4/m	1.0–4.0	109	[54]
α-Ag_1.22_Mn_8_O_16_ (hollandite)	I4/m	1.3–3.8	247	[62]
β-MnO_2_	P4_2_/mnm	1.0–4.3	264–280	[64]
MnO_2_ (amorphous)	-	1.5–4.0	139	[65]
NaMnPO_4_ Mg-doped (olivine)	Pmnb	2.1–4.6	100	[87]
NaMnPO_4_ (maricite)	Pnma	1.5–4.5	102	[90]
Na_3_MnCO_3_PO_4_	P21/m	2.0–4.5	177	[101]
Na_2_MnSiO_4_	Pn	2.0–4.3	210	[122]
Na_4_MnV(PO_4_)_3_ (NASICON)	R-3c	2.5–3.8	112.3	[118]
Na_3_MnTi(PO_4_)_3_ (NASICON)	R-3c	2.4–4.2	114	[112]
Na_3_MnZr(PO_4_)_3_ (NASICON)	R-3c	2.5–4.3	105	[111]
Na_2_MnPO_4_F	P2_1_/n	1.5–4.5	102.4	[96]
Na_2_Fe_0.5_Mn_0.5_PO_4_F	P2_1_/n	2.0–4.5	107	[95]
Na_2_Mn[Mn(CN)_6_] (Prussian blue analog)	P2_1_/n	1.3–4.0	209	[128]
Mn_3_O_4_	I4_1_/amd	0.0–3.0	100–250	[155]
Mn_2_O_3_	Ia-3	0.0–3.0	100–210	[156]
γ-MnOOH	P21/c	0.005–2.8	300–421	[157]
MnFe_2_O_4_@C (cubic spinel and carbon nanofibers)	Fd-3m	0.0–3.0	305	[159]
MnFe_2_O_4_@rGO (cubic spinel and graphene)	Fd-3m	0.0–3.0	258	[158]
CoMn_2_O_4_ (spinel)	Fd-3m	0–2.5	185–347	[160]
